# Neuroimaging Studies of Bipolar Disorder With Suicide Attempts: A Systematic Review

**DOI:** 10.31083/AP50801

**Published:** 2026-09-10

**Authors:** Xiaochao Yu, Zhiqiu He, Kewu Wang, Huina Tang, Yunrong Lu, Jibo Hu

**Affiliations:** ^1^Department of Radiology, The Fourth Affiliated Hospital of School of Medicine, and International School of Medicine, International Institutes of Medicine, Zhejiang University, 322000 Yiwu, Zhejiang, China; ^2^Department of Psychiatry, The Fourth Affiliated Hospital of School of Medicine, and International School of Medicine, International Institutes of Medicine, Zhejiang University, 322000 Yiwu, Zhejiang, China; ^3^Department of Psychiatry, The Second Affiliated Hospital, Zhejiang University School of Medicine, 310009 Hangzhou, Zhejiang, China

**Keywords:** bipolar disorder, diffusion tensor imaging (DTI), magnetic resonance imaging (MRI), suicide, suicide, attempted

## Abstract

**Background:**

Suicide attempts (SA) represent a high-risk indicator for subsequent suicidal behavior in patients with bipolar disorder (BD). However their underlying neurobiological mechanisms remain incompletely understood.

**Method:**

This systematic review synthesized neuroimaging findings from the PubMed and Web of Science databases (January 1990 to April 2026) to elucidate the structural and functional brain alterations associated with SA in BD.

**Results:**

Structural magnetic resonance imaging (MRI) studies revealed reductions in prefrontal-limbic gray matter, alterations in the corpus callosum, and inconsistent findings regarding cerebellar volume. Diffusion tensor imaging (DTI) demonstrated microstructural disconnection in the uncinate fasciculus, inferior fronto-occipital fasciculus, cingulum bundle, forceps minor, and the corpus callosum, with reduced fractional anisotropy correlating with impulsivity and suicidal intent. Functional MRI identified a multilevel pattern of dysfunction, characterized by limbic and paralimbic hyperactivity (including the anterior cingulate cortex, insula, and temporal regions) alongside prefrontal hypoactivity. Network dysregulation was characterized by hyperconnectivity within the default mode network and hypoconnectivity within the salience and executive control networks, as well as altered amygdala-prefrontal and frontotemporal connectivity. Whole-brain dynamic analyses further demonstrated prolonged dwell time in a maladaptive brain state.

**Conclusions:**

Convergent multimodal evidence supports an integrated model in which structural and functional vulnerabilities disrupt emotion regulation and behavioral control, thereby facilitating the transition from suicidal ideation to suicidal behavior. Neuroimaging findings may represent potential candidate biomarkers; however, prospective validation is required before these markers can inform risk stratification or targeted interventions for suicide prevention in BD.

## Main Points

1. Prefrontal-limbic gray-matter reductions and white-matter microstructural disconnection are key structural correlates of suicide attempts in bipolar disorder.

2. Reduced fractional anisotropy in the corpus callosum and fronto-limbic tracts correlates with higher impulsivity and suicidal intent.

3. Functional abnormalities include limbic hyperactivity, default mode network hyperconnectivity, and salience/executive network hypoconnectivity.

4. Destabilized whole-brain dynamics and prolonged dwell time in a maladaptive functional state may facilitate the transition from suicidal ideation to action.

## 1. Introduction

Suicidal behavior represents a serious concern across various psychiatric disorders, with particularly elevated rates observed in bipolar disorder (BD) [1]. Epidemiological data have indicated that the risk for suicide among patients with bipolar disorder can be up to 20 to 30 times that of the general population [2]. Suicide attempts (SA) refer to an individual engaging in non-fatal self-injurious behavior with the intent to die, representing a critical component within the spectrum of suicidal behaviors [3]. Since a previous SA is one of the strongest and most well-established clinical risk factors for predicting future suicide [4], the elucidation of underlying neural substrates (neurobiological mechanisms) is critical for effective prevention [5]. Therefore, identifying biomarkers that can assess the risk of SA and reveal underlying neural mechanisms represents a key objective in current psychiatric and translational neuroscience research.

In recent years, neuroimaging studies have provided significant insights, revealing specific abnormalities in brain structure and function associated with SA in patients with BD (Fig. 1). Structural magnetic resonance imaging (sMRI) studies, focusing on gray matter volume, cortical thickness, and white-matter integrity, have identified convergent abnormalities in bipolar disorder (BD) patients with a history of SA [6,7,8]. The most consistent structural alterations are localized to the prefrontal and limbic systems [9]. Specifically, reduced gray matter volume in the ventromedial and orbitofrontal cortices is a replicated finding, suggesting a stable neural trait linked to suicide risk that was observable in both adolescent and adult samples [10,11]. Parallel investigations of white matter via diffusion tensor imaging (DTI) further indicated microstructural disintegration within pathways connecting the prefrontal and limbic regions, including the corpus callosum, that correlated with suicide risk [12]. Functional magnetic resonance imaging (fMRI) research has elucidated dynamic abnormalities in brain activity and connectivity associated with SA in BD [3]. Resting-state studies have revealed a characteristic imbalance among major brain networks, notably featuring heightened connectivity within the default mode network and weakened connectivity between that network and the salience and central executive networks [13,14]. Task-based fMRI consistently has shown a dysregulated emotion-processing circuit, typified by hyperactivation of the amygdala and insula coupled with diminished regulatory responses from the ventral prefrontal cortex [15,16]. Furthermore, recent studies on dynamic functional connectivity have reported reduced temporal variability in networks involving the anterior cingulate cortex and insula, indicating a more rigid and less adaptive brain state in suicide attempters [17].

**Fig. 1.**
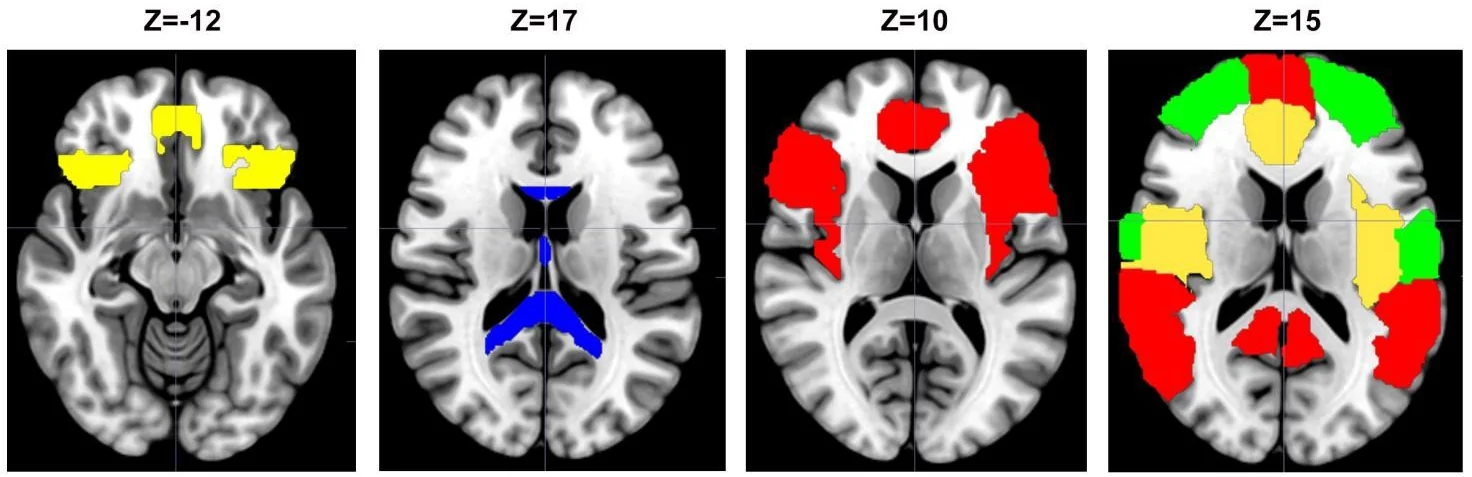
**Abnormal brain regions in bipolar disorder patients with suicide attempts across multiple modalities**. The leftmost panel (structural magnetic resonance imaging [sMRI]) shows reduced gray matter volume in the ventromedial and orbitofrontal regions. The second panel (diffusion tensor imaging [DTI]) reveals abnormalities in the corpus callosum. The third panel (task-functional magnetic resonance imaging [fMRI] and dynamic magnetic resonance imaging [MRI]) displays dysregulation in the ventral prefrontal cortex, amygdala, insula, and anterior cingulate cortex. The fourth panel (resting-state fMRI) illustrates aberrant connectivity within and between brain networks: the default mode network (red), central executive network (green), and salience network (yellow).

This systematic review includes all neuroimaging studies of BD with SA since the establishment of the repository, and summarizes the main findings of 37 articles, 10 of which were published since 2023. Unlike previous reviews that primarily cataloged modality-specific abnormalities separately, this review further integrated multimodal neuroimaging findings into a coherent ‘vulnerability-to-dyscontrol’ cascade model, providing a conceptual framework to inform future biomarker development and targeted interventions [18,19]. This review included a search to uncover neuroimaging patterns characteristic of, and identify potential candidate biomarkers specifically in, patients with BD accompanied by SA. These insights may contribute to more comprehensive clinical assessment and targeted therapeutic strategies for these patients and prevention.

## 2. Methods

We performed a systematic review according to a protocol that used Preferred Reporting Items for Systematic Reviews and Meta-Analyses (PRISMA) statement guidelines (The PRISMA 2020 Statement: An Updated Guideline for Reporting Systematic Reviews, see Fig. 2) (see **Supplementary Material**).

**Fig. 2.**
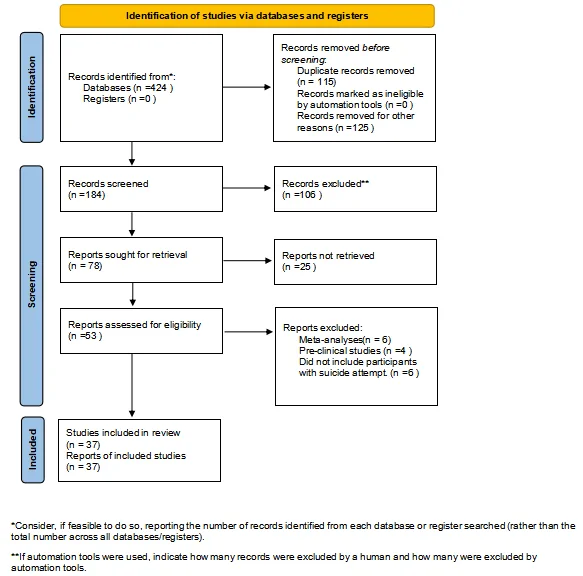
**Preferred Reporting Items for Systematic Reviews and Meta-Analyses (PRISMA) 2020 flow diagram outlining study identification, screening, eligibility assessment, and inclusion for the systematic review of neuroimaging studies on bipolar disorder with suicide attempts**.

### 2.1 Search Strategy

We searched PubMed (https://pubmed.ncbi.nlm.nih.gov/) and Web of Science (http://www.webofscience.com/) for articles published between January 1990 and April 2026 by using the following keywords: (“bipolar disorder” OR “bipolar affective psychosis” OR “bipolar depress*” OR “mani*”) AND “suicid*” AND (“MRI” OR “fMRI” OR “functional magnetic resonance imaging” OR “diffusion tensor imaging” OR “diffusion weighted imaging”). We also searched the reference lists to find potential articles to include. The latest publication date for inclusion was April 18, 2026.

### 2.2 Eligibility Criteria

The eligibility criteria were as follows: (1) participants with BD and a history of SA; (2) studies using MRI methods (including structural, functional, or diffusion MRI); (3) inclusion of a BD group without SA, or a healthy control group, or another clinical group (i.e., MDD); (4) quantitative studies with multiple participants (i.e., no case studies), published in English in peer-reviewed journals.

SA, as defined in this review, included both self-injurious behavior and the intent to die, which allowed for differentiation from suicide ideation (SI) and non-suicidal self-injury (NSSI). Accordingly, studies were excluded if they included only populations with SI or others that did not meet the aforementioned definition. In addition, studies without any control group (e.g., BD without SA or healthy controls) were also excluded. Additional exclusion criteria comprised qualitative designs, preclinical studies, dissertations, meta-analyses, or systematic reviews.

### 2.3 Study Selection and Data Extraction

Two reviewers (Yu and Tang) screened all retrieved records independently. Disagreements regarding the inclusion or exclusion of a study were resolved by consensus; if consensus could not be reached, a third reviewer (He) was consulted to adjudicate. Fig. 2 presents the PRISMA study selection flowchart based on the eligibility criteria. A total of 424 records were identified from database searches. After removing 115 duplicate records and 125 records for other reasons (e.g., irrelevant topic, non-English language), 184 records remained for title and abstract screening. Of these, 106 records were excluded because they did not meet the eligibility criteria (e.g., did not include participants with BD and SA, did not use MRI, or were dissertations or systematic reviews). Two independent reviewers conducted the screening process, achieving a 97.8% agreement rate. The full texts of the remaining 78 reports were sought for retrieval, but 25 reports could not be obtained. The remaining 53 reports were assessed for eligibility, and their reference lists were also screened to identify any additional relevant studies. Of these, 16 reports were excluded for the following reasons: meta-analyses (*n* = 6), preclinical studies (*n* = 4), and studies that did not include participants with an SA (*n* = 6). The same two independent reviewers performed the full-text review, with 100% agreement on the included studies. The final review included 37 studies that met all eligibility criteria.

For each included study, we extracted and documented the following information: (a) authors and year; (b) study design; (c) sample size and diagnosis; (d) mean age (standard deviation) and sex of participants; (e) the subtype of BD and mood state; (f) comorbidity; (g) description of SA; (h) medication status; (i) results of main findings; (j) covariates used; (k) multiple-comparisons correction. This information is presented in Table 1 (Ref. [5,6,7,8,9,20,21,22,23,24,25,26,27,28,29,30,31,32,33,34,35,36,37,38,39,40,41,42,43,44,45,46,47,48,49,50,51]) and **Supplementary Table 1**.

**Table 1.** **Neuroimaging study characteristics and data extraction**.

Authors and year	Study design	Sample size and diagnosis	Mean age (standard deviation)	Sex	The subtype of bipolar disorder and mood state of patients	Comorbidity	Results of main findings
Huang et al. (2023) [20]	sMRI	108 subjects: 26 BD with SA 26 BD with NSA 56 HCs	34.41 (8.07) SA: 34.3 (8.3) NSA: 35.6 (9.0) HC: 33.9 (7.6)	BD: 27 F, 25 M HC: 34 F, 22 M	/ euthymic state	/	↑ BIS scores correlated with ↑ GMV in the left pars triangularis and ↑ surface area in the left pars opercularis. Compared to NSA, BD patients with SA exhibited ↑ GMV in the inferior frontal gyrus.
Villa et al. (2023) [7]	sMRI	201 subjects: 29 BD with SA 74 BD with NSA 98 HCs	51.07 (7.01) BD: 51.7 (7.9) HC: 50.3 (6.0)	BD: 59 F, 44 M HC: 50 F, 48 M	88 BD-I, 15 BD-II 36 depression, 49 euthymic state, 12 elevated, 6 mixed	vascular, endocrine, PTSD, substance-related disorders, alcohol-related disorder, eating disorder, obesessive compulsive disorder, panic disorder, general anxiety disorder, social phobia, specific phobia	BD exhibited ↓ cortical thickness in the prefrontal, cingulate, sensorimotor, parahippocampal, insular, temporal, parietal, and occipital regions, as well as ↓ gray matter volume in the hippocampus, amygdala, thalamus, and striatum. A history of suicide attempt was linked to thinner dorsolateral prefrontal cortex.
Jabbi et al. (2020) [21]	sMRI	121 subjects: 23 BD with SA 58 BD with NSA 40 HCs	21.38 (5.63) 42 MANIC: 18.45 (4.68) 39 DEPRESSED: 23.43 (5.59) HC: 22.45 (5.44)	BD: 53 F, 28 M HC: 21 F, 19 M	BD-I 42 mania, 39 depression	/	↓ GMV in the subgenual cingulate and dorsolateral prefrontal cortices in bipolar disorder patients compared to controls.
Huber et al. (2019) [22]	sMRI	58 subjects: 15 BD with SA 18 BD with NSA 25 HCs	17.93 (2.47) SA: 17.47 (2.45) NSA: 17.44 (2.48) HC: 18.56 (2.42)	BD: 23 F, 10 M HC: 16 F, 9 M	BD-I, BD-II, NOS depression	/	Bipolar youth with SA showed ↓ left lateral orbitofrontal cortex (OFC) volumes versus controls. Controls and BD without attempters had ↑ OFC cortical thickness than BD attempters.
Gifuni et al. (2017) [23]	sMRI	209 subjects: 72 MDD 64 BD (61 SA, 75 NSA)/136 patients 73 HCs	38.71 (8.93) MDD: 37.7 (9.1) BD: 39.3 (10.6) HC: 39.2 (7.0)	MDD: 52 F, 20 M BD: 39 F, 25 M HC: 29 F, 44 M	/ euthymic state	/	↓ in the volume of the mid-anterior, central, and mid-posterior corpus callosum in bipolar patients, independent of suicidality.
Duarte et al. (2017) [24]	sMRI	59 subjects: 20 BD with SA 19 BD with NSA 20 HCs	40.22 (11.54) SA: 41.10 (12.64) NSA: 42.26 (11.70) HC: 37.40 (10.20)	BD: 23 F, 16 M HC: 11 F, 9 M	BD-I remission (not meeting any criteria for a mood episode within the last 2 months)	16 BD with psychiatric comorbidities	In BD-I patients with SA, GMV was significantly ↑ in the right rACC; this increase was more pronounced and spread further into the left ACC among the high-lethality subgroup. Moreover, GMV in the insula and orbitofrontal cortex was also associated with suicide lethality.
Lijffijt et al. (2014) [25]	sMRI	93 subjects: 51 BD with SA 42 BD with NSA	38.63 (11.14) NSA: 41.0 (11.3) SA: 36.6 (10.7)	BD: 93 F, 0 M	65 BD-I, 28 BD-II 46 depression, 4 mania, 14 mixed, 29 euthymic	/	PFCGM volume was ↓ in patients with SA versus those without SA among those with past psychiatric hospitalization. PFCGM Volume was ↑ in patients with SA versus those without SA among those without hospitalization. ↑ frontal pole volume and younger age at first hospitalization predicted multiple SA.
Nery-Fernandes et al. (2012) [26]	sMRI	62 subjects: 19 BD with SA 21 BD with NSA 22 HCs	39.80 (11.34) SA: 39.8 (11.4) NSA: 42.0 (8.6) HC: 37.7 (13.5)	BD: 29 F, 11 M HC: 12 F, 10 M	BD-I euthymic state	14 BD with psychiatric comorbidities	↓ in the genu and isthmus areas of the corpus callosum in bipolar patients.
Baldaçara et al. (2011) [27]	sMRI	62 subjects: 20 BD with SA 20 BD with NSA 22 HCs	40.91 (10.02) BD: 41.10 (11.50) HC: 37.72 (13.63)	BD: 29 F, 11 M HC: 12 F, 10 M	BD-I euthymic state	no comorbidity	Right cerebellum, left cerebellum, and vermis were ↓ in the BD group; BD subjects with and without SA showed no volumetric differences.
Matsuo et al. (2010) [28]	sMRI	47 subjects: 10 BD with SA 10 BD with NSA 27 HCs	38.30 (12.96) SA: 36.2 (10.1) NSA: 44.2 (12.5) HC: 36.9 (13.8)	BD: 20 F, 0 M HC: 27 F, 0 M	/	/	Suicidal BD group had the smallest areas in any regional corpus callosum area.
Vishwakarma et al. (2025) [29]	DTI	32 BD 16 BD with SA 16 BD with NSA	35.25 (11.89) SA: 34.3 (10.4) NSA: 36.2 (13.5)	SA: 6 F, 10 M NSA: 9 F, 7 M	BD-I remission and currently euthymic	/	In the SA group, FA values indicated ↓ white matter anisotropy across five clusters: the bilateral inferior fronto-occipital fasciculus (IFOF), left superior longitudinal fasciculus (SLF), cingulate, and forceps minor.
Jiang et al. (2022) [8]	DTI	64 subjects: 14 BD with SA 24 BD with NSA 26 HCs	27.13 (6.15) SA: 26.79 (7.68) NSA: 26.13 (1.91) HC: 28.23 (7.69)	BD: 20 F, 18 M HC: 16 F, 10 M	19 BD-I, 13 BD-II, 6 unclear 15 depression, 8 mania, 15 remission	/	Relative to BD with NSA and HCs, BD with SA showed ↓ FA in the bilateral middle cerebellar peduncle and left superior corona radiata. Both BD with SA and BD with NSA had ↓ FA in the bilateral body and genu of the CC than HCs.
Lima Santos et al. (2021) [30]	DTI	108 subjects: 20 BD with SA 48 BD with NSA 40 HCs	26.14 (4.26) SA: 27.7 (3.6) NSA: 25.9 (4.0) HC: 25.7 (4.7)	BD: 33 F, 35 M HC: 19 F, 21 M	/	anxiety disorder, psychotic disorders, substance use disorders, personality disorders, ADHD	In the SA group, fractional anisotropy was reduced in the middle part of the FMIN, as well as in the anterior and posterior parts of the right CB. Furthermore, FMIN abnormalities were linked to suicidal ideation and the levels of emotional distress at the time of scanning.
Wei et al. (2020) [31]	DTI	199 subjects: 27 MDD with SA 49 MDD with NSA 25 BD with SA 49 BD with NSA 49 HCs	29.08 (8.68) MDD with SA: 28.04 (11.06) MDD with NSA: 30.03 (0.91) BD with SA: 27.08 (8.0) BD with NSA: 27.69 (9.97) HC: 31.12 (9.95)	MDD: 56 F, 20 M BD: 41 F, 33 M HC: 31 F, 18 M	/ 94 depression, 20 mania, 36 remitted/stable	/	BD with prior SA showed ↓ FA in the bilateral IFOF, bilateral UF, CC, and WM bundles. MDD with prior SA exhibited the lowest mean FA values in the bilateral thalami.
Reich et al. (2019) [32]	DTI	42 subjects: 12 BD with SA 18 BD with NSA 12 HCs	36.63 (12.75) SA: 36.9 (14.9) NSA: 38.8 (11.7) HC: 33.1 (12.3)	BD: 21 F, 9 M HC: 9 F, 3 M	27 BD-I, 3 BD-II /	alcohol use disorder, cannabis use disorder, sedative/hypnotic/anxiolytic use disorder, bulimia nervosa, eating disorder not otherwise specified, panic disorder with agoraphobia, PTSD, social phobia, obsessive-compulsive disorder, generalized anxiety disorder, polysubstance use disorder	In BD with SA patients, ↑ FA in anterior brain regions was associated with higher overall impulsivity as measured by the UPPS-P Impulsive Behavior Scale.
Cyprien et al. (2016) [33]	DTI	121 subjects: 25 BD with SA 16 BD with NSA 50 MDD 30 HCs	37.18 (8.62) BD: 37.7 (9.1) MDD: 36.5 (8.8) HC: 37.6 (7.8)	121 F, 0 M	32 BD-I, 9 BD-II euthymic state	/	Compared to MDD patients and HC, BD-NSA showed ↓ FA in the CC genu and body. BD-SA exhibited ↓ FA in all CC regions versus HC. Elevated Suicidal Intent Scale scores correlated with marked splenium alterations.
Mahon et al. (2012) [34]	DTI	44 subjects: 14 BD with SA 15 BD with NSA 15 HCs	34.53 (12.77) SA: 33.3 (14.1) NSA: 36.5 (12.3) HC: 33.7 (12.6)	BD: 11 F, 18 M HC: 7 F, 8 M	BD-I /	substance use disorder, anorexia nervosa, panic disorder with/without a history of agora phobia, anxiety disorder not otherwise specified, PTSD, generalized anxiety disorder, specific phobia, and bulimia nervosa, obsessive–compulsive disorder, agoraphobia without a history of panic disorder	Compared to BD non-attempters and HC, BD patients with SA showed ↓ FA in the left orbital frontal white matter and ↑ overall impulsivity.
Huang et al. (2025) [35]	fMRI	127 subjects: 34 BD with SA 38 BD with NSA 55 HCs	35.33 (9.24) SA: 35.0 (10.0) NSA: 37.1 (10.7) HC: 34.3 (7.5)	BD: 38 F, 34 M HC: 32 F, 23 M	/ euthymic state	/	Compared to BDNSA, BDSA exhibited ↓ FC between the PFC and sensorimotor regions (postcentral and precentral gyri) as well as thalamic areas.
Zhong et al. (2024) [36]	fMRI	114 subjects: 39 BD with SA 40 BD without SA 35 HCs	23.79 (5.69) SA: 22.67 (4.65) NSA: 24.17 (5.91) HC: 24.60 (6.44)	BD: 51 F, 29 M HC: 19 F, 16 M	/ depression	/	Relative to NSA BD patients and HCs, BD with SA showed ↑ dFC variability between the right caudal hippocampus and the left superior temporal gyrus (STG).
Wei et al. (2024) [37]	fMRI	191 subjects: 61 BD with SA 62 BD with NSA 68 HCs	26.48 (5.19) SA: 25.72 (6.07) NSA: 26.03 (4.79) HC: 27.56 (4.55)	BD: 83 F, 40 M HC: 44 F, 24 M	/ depression	/	Suicide-related patterns were characterized by hyper-connectivity within the DMN and hypo-connectivity within the SN and CEN.
Wang et al. (2024) [38]	fMRI	195 subjects: 50 BD with SA 64 BD with NSA 81 HCs	29.63 (8.75) SA: 27.72 (8.5) NSA: 30.03 (9.64) HC: 30.49 (8.06)	BD: 72 F, 42 M HC: 54 F, 27 M	BD-I, BD-II major depression episode	no other mental disorders	Relative to NSA, SA patients exhibited ↑ fractional window and ↑ dwell time in the suicide-related state, as well as ↑ number of state transitions.
Vai et al. (2023) [39]	fMRI	106 subjects: 17 BD with SA 57 BD with NSA 32 HCs	44.96 (11.50) SA: 46.65 (8.62) NSA: 47.03 (10.95) HC: 40.37 (12.71)	BD: 57 F, 17 M HC: 15 F, 17 M	74 BD-I major depression episode	/	In SA, ↓ habituation was observed in multiple cortico-limbic regions: the amygdalae, cingulate and parietal cortex, insula, hippocampus, para-hippocampus, cerebellar vermis, thalamus, and striatum. Additionally, ↓ habituation rates in the amygdala correlated with more severe current depressive and suicidal symptoms.
Tian et al. (2023) [40]	fMRI	288 subjects: 75 BD with SA 101 BD with NSA 112 HCs	30.63 (9.82) SA: 27.11 (9.00) NSA: 31.00 (10.35) HC: 32.65 (9.28)	BD: 111 F, 65 M HC: 71 F, 41 M	/ major depression episode	/	↑ dynamic ALFF values in the right anterior cingulate cortex, left thalamus, and right precuneus were found in BD with SA.
Shunkai et al. (2023) [41]	fMRI	103 subjects: 30 BD with SA 38 BD with NSA 35 HCs	23.81 (5.85) SA: 22.50 (4.65) NSA: 24.11 (6.10) HC: 24.60 (6.44)	BD: 44 F, 24 M HC: 19 F, 16 M	BD-II depression	/	In the SA group, ↓ dFC variability was observed between the left dorsal anterior insula and the left anterior cerebellum.
Sankar et al. (2022) [42]	fMRI	140 subjects: 40 BD with SA 49 BD with NSA 51 HCs	31.01 (11.62) SA: 26.7 (8.5) NSA: 30.9 (12.0) HC: 34.5 (12.4)	BD: 60 F, 29 M HC: 31 F, 20 M	BD-I, BD-II 36 euthymic, 25 depression, 28 elevated	substance use disorder, PTSD, panic disorder, social phobia, generalized anxiety disorder, specific phobia, obsessive-compulsive disorder, anorexia nervosa, eating disorders NOS, binge eating disorder, bulimia nervosa	In the bilateral ventromedial prefrontal cortex (vmPFC) and right anterior insula (RaIns), ICD was ↓ in SAs compared to NSAs and HCs. Seed connectivity analyses showed altered FC originating from vmPFC to the bilateral anteromedial orbitofrontal cortex, left ventrolateral PFC (vlPFC), and cerebellum, as well as from RaIns to the right vlPFC and temporopolar cortices.
Zhong et al. (2022) [43]	fMRI	103 subjects: 30 BD with SA 38 BD with NSA 35 HCs	23.81 (5.85) SA: 22.50 (4.65) NSA: 24.11 (6.10) HC: 24.60 (6.44)	BD: 44 F, 24 M HC: 19 F, 16 M	BD-II depression	/	In SA, ↑ dFC variability was observed between the right vCa and the right insula, which negatively correlated with processing speed. In NSA, ↓ dFC variability between the left dlPu and the right postcentral gyrus was found, positively correlating with reasoning and problem-solving. Additionally, ↓ dFC variability was present in both SA and NSA between the right dCa and the left MTG, as well as between the right dlPu and the right calcarine.
Zhang et al. (2022) [44]	fMRI	65 subjects: 24 BD with SA 15 BD with NSA 26 HCs	32.93 (11.47) SA: 33.5 (12.1) NSA: 29.8 (10.9) HC: 34.2 (11.3)	BD: 24 F, 15 M HC: 18 F, 8 M	BD-I major depression episode	/	(SA+NSA) showed ↓ FC between the bilateral thalamus and frontal cortex, with the most pronounced reduction in the SA group. Patients exhibited ↓ FC values between the bilateral inferior frontal gyrus and both the inferior temporal gyrus and fusiform gyrus, and this FC was stronger in SA than in NSA. Additionally, (SA+NSA) demonstrated ↓ FC involving the bilateral amygdala, medial frontal cortex, and ACC.
Wang et al. (2022) [45]	fMRI	178 subjects: 46 BD with SA 61 BD with NSA 71 HCs	30.13 (9.14) SA: 27.04 (8.10) NSA: 30.57 (10.05) HC: 31.75 (8.57)	BD: 66 F, 41 M HC: 47 F, 24 M	BD-I, BD-II major depression episode	no other mental disorders	In SA patients, the suicide-related functional state was characterized by ↓ dwell time and ↑ FC strength between the right ACC and subcortical (SubC) network regions. Additionally, SA patients showed ↑ number of state transitions.
Tian et al. (2021) [5]	fMRI	159 subjects: 42 BD with SA 57 BD with NSA 60 HCs	28.97 (8.34) SA: 27.31 (8.28) NSA: 29.26 (9.26) HC: 30.83 (7.34)	BD: 60 F, 39 M HC: 38 F, 22 M	BD-II major depression episode	substance abuse/dependence	In BD-II patients with SA, brain activity and dALFF were ↑ in the anterior cingulate cortex.
Gong et al. (2020) [46]	fMRI	156 subjects: 20 BD with SA 35 BD with SI 51 BD with NSI 50 HCs	26.63 (8.68) SA: 27.35 (8.29) SI: 23.74 (6.97) NSI: 26.82 (9.64) HC: 28.16 (8.63)	BD: 51 F, 55 M HC: 29 F, 21 M	BD-II depression	/	Across the three bipolar disorder II groups, dynamic ALFF was markedly ↓ in the bilateral precuneus/posterior cingulate cortex. In the SA group, dynamic ALFF was ↑ in the right superior temporal gyrus, right inferior temporal gyrus, and the right temporal pole.
Cheng et al. (2021) [47]	fMRI	179 subjects: 30 BD with SA 82 BD with NSA 67 HCs	24.16 (6.69) SA: 23.53 (5.37) NSA: 24.37 (6.87) HC: 24.18 (7.06)	BD: 71 F, 41 M HC: 37 F, 30 M	BD-I /	no comorbidity	Between SA and NSA, we identified regional differences in GBC values in emotion-encoding circuits, which included the left superior temporal gyrus, bilateral insula/rolandic operculum, and right precuneus (PCu)/cuneus.
Wang et al. (2020) [48]	fMRI	167 subjects: 38 BD with SA 60 BD with NSA 69 HCs	30.62 (9.35) SA: 27.53 (8.28) NSA: 31.07 (10.45) HC: 31.94 (8.61)	BD: 60 F, 38 M HC: 47 F, 22 M	BD-II major depression episode	BD with substance abuse/dependence	In SA patients, ↓ functional connectivity was found between limbic/subcortical and frontoparietal networks. ↓ nodal strength was observed in left head of the caudate nucleus (HCN), raphe nucleus (RN), right nucleus accumbens (NAcc), and right subgenual anterior cingulate cortex (sgACC) while ↓ nodal efficiency was seen in right sgACC and right HCN. RN nodal strength and right sgACC nodal efficiency also significantly negatively correlated with NGASR scores.
Shaffer et al. (2022) [49]	fMRI quantitative T1ρ	39 subjects: 19 BD with SA 20 BD with NSA	39.30 (13.40) SA: 46.8 (11.9) NSA: 31.0 (10.5)	SA: 10 F, 9 M NSA: 6 F, 14 M	BD-I 16 enthymia, 9 depression, 14 mania	/	In SA, fMRI task-related activation was ↑ in visual areas and the cerebellum. The number of SAs correlated with differences in BOLD response within the amygdala, prefrontal cortex, and cerebellum. Additionally, ↑ quantitative T1 rho was associated with the number of suicide attempts in limbic, basal ganglia, and prefrontal cortex regions.
Zhao et al. (2021) [6]	sMRI fMRI	220 subjects: 40 BD with SA 70 BD with NSA 110 HCs	28.14 (8.02) SA: 28.15 (7.18) NSA: 28.26 (8.11) HC: 28.05 (8.32)	SA: 24 F, 16 M NSA: 44 F, 26 M HC: 74 F, 36 M	/ 45 depression, 26 mania, 39 remitted/stable	/	In both BD groups, gray matter volumes were ↓ and ALFF was ↑ in the medial, ventral, and dorsolateral PFC. In the BD+SA group, ALFF was ↓ in the mPFC, vPFC, and DLPFC.
Jiang et al. (2020) [50]	DTI fMRI	288 subjects: 75 BD with SA 101 BD with NSA 112 HCs	30.63 (9.82) SA: 27.11 (9.00) NSA: 31.00 (10.35) HC: 32.65 (9.28)	BD: 111 F, 65 M HC: 71 F, 41 M	/ major depression episode	BD with substance abuse/dependence	Compared to NSA, BD patients with SA exhibite ↓ structural connectivity in central-temporal regions, ↑ functional connectivity in frontal-temporal regions, and also ↓ structural-functional coupling.
Fan et al. (2019) [51]	sMRI DTI	83 subjects: 21 BD with SA 25 BD with NSA 19 MDD with SA 18 MDD with NSA	19.60 (3.10) BD with SA: 20.3 (3) BD with NSA: 20.2 (3.4) MDD with SA: 18.7 (3) MDD with NSA: 18.9 (2.7)	BD: 39 F, 7 M MDD: 31 F, 6 M	/	substance abuse, panic disorder, PTSD, social phobia, specific phobia, obsessive-compulsive, anorexia nervosa, bulimia nervosa, attention deficit/hyperactivity	Compared to NSAs, SAs differed in ventral PFC GM volume and fronto-limbic WM FA. Disorder-specific differences included altered dlPFC and hippocampal GM volume and UF FA in BD SAs, and altered dorsomedial and dlPFC GM volume and dorsal frontal WM in MDD SAs.
Johnston et al. (2017) [9]	sMRI DTI fMRI	68 subjects: 26 BD with SA 42 BD with NSA	20.56 (3.10) SA: 20.5 (3.0) NSA: 20.6 (3.2)	SA: 20 F, 6 M NSA: 23 F, 19 M	/ 34 euthymic state, 16 depression, 18 elevated	substance use disorders, panic disorder, PTSD, social phobia, specific phobia, obsessive-compulsive disorder, generalized anxiety disorder, anorexia nervosa, bulimia nervosa, eating disorder not otherwise specified, attention deficit hyperactivity disorder	The attempter group exhibited ↓ gray matter volume in the orbitofrontal cortex, hippocampus, and cerebellum, as well as ↓ white matter integrity in the uncinate fasciculus, ventral frontal, and right cerebellum regions; and amygdala functional connectivity to the left ventral and right rostral prefrontal cortex. Among attempters, there was a significant negative correlation between right rostral prefrontal connectivity and suicidal ideation and between left ventral prefrontal connectivity and attempt lethality.

↑, increase; ↓, decrease; M, male; F, female; MRI, magnetic resonance imaging; BD, bipolar disorder; SA, suicide attempt; NSA, non-suicide attempt; SI, suicidal ideation; HC, healthy control; BD-I, bipolar I disorder; BD-II, bipolar II disorder; GMV, gray matter volume; MDD, major depressive disorder; WMH, white matter hyperintensities; OFC, orbitofrontal cortex; CC, corpus callosum; PFCGM, prefrontal cortex gray matter; BIS, Barratt impulsiveness scale; UMDD, unipolar major depressive disorder; PVH, periventricular hyperintensities; rACC, rostral anterior cingulate cortex; PFC, prefrontal cortex; GM, gray matter; WM, white matter; FA, fractional Anisotropy; UF, uncinate fasciculus; DLPFC, dorsolateral prefrontal cortex; FMIN, forceps minor; CB, cingulum bundle; MRS, magnetic resonance spectroscopy; Cho, choline-containing compounds; Cr, creatine; NAA, N-acetylaspartate; LN, lentiform nucleus;IFOF, inferior fronto-occipital fasciculus; dACC, dorsal anterior cingulate cortex; ALFF, amplitude of low-frequency fluctuations; fALFF, fractional amplitude of low-frequency fluctuations; dALFF, dynamic amplitude of low-frequency fluctuations; rsFC, resting-state functional connectivity; ICD, intrinsic connectivity distribution; dFC, dynamic functional connectivity; vCa, ventral caudate nucleus; dCa, dorsal caudate nucleus; dlPu, dorsolateral putamen; MTG, middle temporal gyrus; GBC, global functional connectivity; DMN, default mode network; SN, salience network; CEN, central executive network; BOLD, blood-oxygen-level-dependent; HCN, head of caudate nucleus; RN, raphe nucleus; NAcc, nucleus accumbens; sgACC, subgenual anterior cingulate cortex; PSA, patient with suicide attempt; PTSD, posttraumatic stress disorder; ADHD, attention-deficit/hyperactivity disorder; NOS, not otherwise specified. Of note, Tian et al. (2023) [40] and Jiang et al. (2020) [50] focused on different metrics for functional imaging, with one investigating dynamic ALFF and the other functional connectivity. Discrepant brain regions were reported in the studies by Shunkai et al. (2023) [41] and Zhong et al. (2022) [43], as well as in those by Nery‑Fernandes et al. (2012) [26] and Baldaçara et al. (2011) [27], and their conclusions can complement each other.

### 2.4 Quality Assessment

The National Institutes of Health (NIH) quality assessment tool [52] for case-control studies was used to assess the methodological quality and risk of bias of the included research. The tool evaluates compliance with 12 items, with each item scored as 1 for “yes”, 0.5 for “no”, and 0 for other responses. A cumulative score was calculated for each included study, and studies were subsequently rated as good, fair, or poor (see Table 2, Ref. [5,6,7,8,9,20,21,22,23,24,25,26,27,28,29,30,31,32,33,34,35,36,37,38,39,40,41,42,43,44,45,46,47,48,49,50,51]). This tool was chosen for its widespread use and sound reliability and validity.

**Table 2.** **The National Institutes of Health (NIH) quality assessment tool ratings**.

Authors (year)	Question (NIH assessment tool)^a^												Rating
	1	2	3	4	5	6	7	8	9	10	11	12	
Baldaçara et al. (2011) [27]	Yes	Yes	No	Yes	Yes	Yes	No	Yes	CD	Yes	No	No	Fair
Cheng et al. (2021) [47]	Yes	Yes	No	Yes	Yes	Yes	No	Yes	CD	Yes	No	Yes	Good
Cyprien et al. (2016) [33]	Yes	Yes	No	Yes	Yes	Yes	No	Yes	CD	Yes	CD	Yes	Fair
Duarte et al. (2017) [24]	Yes	Yes	No	Yes	Yes	Yes	No	Yes	Yes	Yes	No	Yes	Good
Fan et al. (2019) [51]	Yes	Yes	No	Yes	Yes	Yes	No	Yes	Yes	Yes	No	Yes	Good
Gifuni et al. (2017) [23]	Yes	Yes	No	Yes	Yes	Yes	No	Yes	CD	Yes	No	Yes	Fair
Gong et al. (2020) [46]	Yes	Yes	No	Yes	Yes	Yes	No	Yes	Yes	Yes	No	Yes	Good
Huang et al. (2023) [20]	Yes	Yes	No	Yes	Yes	Yes	No	Yes	CD	Yes	No	Yes	Fair
Huang et al. (2025) [35]	Yes	Yes	No	Yes	Yes	Yes	No	Yes	CD	Yes	No	Yes	Good
Huber et al. (2019) [22]	Yes	Yes	No	Yes	Yes	Yes	No	Yes	CD	Yes	No	Yes	Good
Jabbi et al. (2020) [21]	Yes	Yes	No	Yes	Yes	Yes	No	CD	Yes	Yes	No	No	Fair
Jiang et al. (2020) [50]	Yes	Yes	No	Yes	Yes	Yes	No	Yes	Yes	Yes	No	Yes	Good
Jiang et al. (2022) [8]	Yes	Yes	No	Yes	Yes	Yes	No	Yes	Yes	Yes	No	Yes	Good
Johnston et al. (2017) [9]	Yes	Yes	No	Yes	Yes	Yes	No	Yes	Yes	Yes	No	Yes	Good
Lijffijt et al. (2014) [25]	Yes	Yes	No	Yes	Yes	Yes	No	Yes	Yes	Yes	CD	Yes	Good
Mahon et al. (2012) [34]	Yes	Yes	No	Yes	Yes	Yes	No	Yes	Yes	Yes	No	Yes	Good
Matsuo et al. (2010) [28]	Yes	Yes	No	Yes	Yes	Yes	No	Yes	CD	Yes	No	Yes	Fair
Nery-Fernandes et al. (2012) [26]	Yes	Yes	No	Yes	Yes	Yes	No	Yes	CD	Yes	No	Yes	Fair
Reich et al. (2019) [32]	Yes	Yes	No	Yes	Yes	Yes	No	Yes	Yes	Yes	No	Yes	Good
Sankar et al. (2022) [42]	Yes	Yes	No	Yes	Yes	Yes	No	Yes	Yes	Yes	No	Yes	Good
Lima Santos et al. (2021) [30]	Yes	Yes	No	Yes	Yes	Yes	No	Yes	CD	Yes	No	Yes	Good
Shaffer et al. (2022) [49]	Yes	Yes	No	Yes	Yes	Yes	No	Yes	Yes	Yes	No	Yes	Good
Shunkai et al. (2023) [41]	Yes	Yes	No	Yes	Yes	Yes	No	Yes	Yes	Yes	No	Yes	Good
Tian et al. (2021) [5]	Yes	Yes	No	Yes	Yes	Yes	No	Yes	Yes	Yes	No	Yes	Good
Tian et al. (2023) [40]	Yes	Yes	No	Yes	Yes	Yes	No	Yes	CD	Yes	No	Yes	Good
Vai et al. (2023) [39]	Yes	Yes	No	Yes	Yes	Yes	No	Yes	Yes	Yes	No	Yes	Good
Villa et al. (2023) [7]	Yes	Yes	No	Yes	Yes	Yes	No	Yes	CD	Yes	No	Yes	Good
Vishwakarma et al. (2025) [29]	Yes	Yes	No	Yes	Yes	Yes	No	Yes	Yes	Yes	No	Yes	Good
Wang et al. (2020) [48]	Yes	Yes	No	Yes	Yes	Yes	No	Yes	Yes	Yes	No	Yes	Good
Wang et al. (2022) [45]	Yes	Yes	No	Yes	Yes	Yes	No	Yes	Yes	Yes	No	Yes	Good
Wang et al. (2024) [38]	Yes	Yes	No	Yes	Yes	Yes	No	Yes	Yes	Yes	No	Yes	Good
Wei et al. (2020) [31]	Yes	Yes	No	Yes	Yes	Yes	No	Yes	Yes	Yes	No	Yes	Good
Wei et al. (2024) [37]	Yes	Yes	No	Yes	Yes	Yes	No	Yes	Yes	Yes	No	Yes	Good
Zhao et al. (2021) [6]	Yes	Yes	No	Yes	Yes	Yes	No	Yes	CD	Yes	No	Yes	Good
Zhong et al. (2022) [43]	Yes	Yes	No	Yes	Yes	Yes	No	Yes	Yes	Yes	No	Yes	Good
Zhong et al. (2024) [36]	Yes	Yes	No	Yes	Yes	Yes	No	Yes	Yes	Yes	No	Yes	Good
Zhang et al. (2022) [44]	Yes	Yes	No	Yes	Yes	Yes	No	Yes	CD	Yes	No	Yes	Good

Source: National Heart, Lung, and Blood Institute; National Institutes of Health; U.S. Department of Health and Human Services. CD, Cannot determine; NA, Not applicable; NIH, National Institutes of Health; NR, Not reported. Yes = 1, No = 0.5, Others = 0. The total score was calculated by summing the scores of all items. Good (9.5–12 points): The study has minimal risk of bias. Fair (6.5–9 points): The study has some risk of bias, but not sufficient to invalidate the results. Poor (0–6 points): The study has a significant risk of bias. ^a^The NIH Quality Assessment Tool for Case–Control Studies includes 12 questions: 1 = Was the research question or objective in this paper clearly stated? 2 = Was the study population clearly specified and defined? 3 = Did the authors include a sample size justification? 4 = Were controls selected or recruited from the same or similar population that gave rise to the cases (including the same timeframe)? 5 = Were the definitions, inclusion and exclusion criteria, algorithms or processes used to identify or select cases and controls valid, reliable, and implemented consistently across all study participants? 6 = Were the cases clearly defined and differentiated from controls? 7 = If less than 100 percent of eligible cases and/or controls were selected for the study, were the cases and/or controls randomly selected from those eligible? 8 = Was there use of concurrent controls? 9 = Were the investigators able to confirm that the exposure/risk occurred prior to the development of the condition or event that defined a participant as a case? 10 = Were the measures of exposure/risk clearly defined, valid, reliable, and implemented consistently (including the same time period) across all study participants? 11 = Were the assessors of exposure/risk blinded to the case or control status of participants? 12 = Were key potential confounding variables measured and adjusted statistically in the analyses? If matching was used, did the investigators account for matching during study analysis?

## 3. Results

We identified 37 articles that met the inclusion criteria. The findings are summarized in Table 1, which is organized by neuroimaging modalities: sMRI, DTI, fMRI, and multi-modal imaging. Publications of the same modality are presented sequentially, from most recent to earliest.

### 3.1 Structural Imaging

#### 3.1.1 Magnetic Resonance Imaging

Ten sMRI reports of BD patients with a history of SA pointed to a multifaceted pattern of alterations, predominantly within frontal-limbic circuits, interhemispheric connectivity pathways, and the cerebellum, with notable interactions with clinical features such as impulsivity and illness severity.

The prefrontal cortex (PFC) emerged as a critical region with complex, subregion-specific changes. A broad pattern of cortical thinning and subcortical gray matter volume (GMV) reduction was observed in BD relative to healthy controls [7], with GMV reductions noted in the medial, ventral, and dorsolateral PFC [6,7]. Within this context, a history of SA was specifically linked to reduced cortical thickness in the dorsolateral PFC (dlPFC) [21]. However, GMV findings within the inferior frontal gyrus (IFG) and its subregions presented a nuanced picture. BD patients with SA demonstrated greater GMV in the right IFG than did non-attempters [20], and higher impulsivity scores (BIS scores) among attempters correlated with increased GMV in the left pars triangularis and surface area in the left pars opercularis [20]. In contrast, the orbitofrontal cortex (OFC) showed consistent reductions associated with suicidal behavior. BD youth with SA exhibited decreased left lateral OFC volumes and thinner OFC cortex than did both healthy controls and BD non-attempters, with OFC volume further correlating negatively with suicide lethality [22]. This pattern extended to the anterior cingulate and insular cortices, where increased GMV in the rostral anterior cingulate cortex (rACC) in BD-I patients with SA—particularly in high-lethality subgroups—and insular/OFC GMV were related to lethality [24]. Additionally, the relationship between prefrontal cortex gray matter (PFCGM) volume and SA may also have been moderated by illness severity, as it varied by history of psychiatric hospitalization: PFCGM volume was lower in patients with SA than in those without SA among those with past psychiatric hospitalization, whereas PFCGM volume was greater in patients with SA than in those without SA among those without hospitalization. Greater frontal-pole volume and younger age at first hospitalization predicted multiple SA events [25]. Further supporting these regional findings, compared to non-attempters, SA patients differed in ventral PFC GM volume, with disorder-specific differences including altered dlPFC and hippocampal GM volume in BD SAs, and altered dorsomedial and dlPFC GM volume in MDD SAs [51]. Moreover, the attempter group exhibited lower GMV in the OFC, hippocampus, and cerebellum [9].

Alterations in white-matter structure, crucial for neural connectivity, were also evident. The corpus callosum (CC), the primary commissure for interhemispheric communication, showed consistent area or volume reductions across its subregions (e.g., genu, isthmus, mid-anterior, central, and mid-posterior) in BD patients [23,26,28]. Although one study suggested that this reduction may occur independently of suicidality [23], another indicated that suicidal BD patients possess the smallest regional CC areas, highlighting it as a potential vulnerability marker [28].

Findings regarding the cerebellum were less consistent in relation to SA. Although BD patients as a group showed lower volumes in the bilateral cerebellum and vermis than did controls [27], studies have not consistently found volumetric differences within BD when comparing those with and without a SA history [27]. Nevertheless, reduced GMV in the cerebellum has been reported in attempters [9].

#### 3.1.2 Diffusion Tensor Imaging

The seven DTI studies investigating white-matter (WM) microstructure in BD patients with a history of SA consistently pointed towards disrupted integrity within critical neural networks, particularly those subserving frontal-limbic connectivity and interhemispheric communication.

A convergent finding across multiple studies was reduced fractional anisotropy (FA)—a marker of WM organization and integrity—within fronto-limbic pathways in BD patients with SA. Specifically, alterations were reported in the inferior fronto-occipital fasciculus (IFOF), uncinate fasciculus (UF), and the cingulum bundle [29,31]. Those tracts are fundamental for connecting prefrontal regulatory regions with limbic and subcortical areas involved in emotion generation. Furthermore, reduced FA has been observed in the forceps minor, a tract interconnecting prefrontal cortices, and in the left orbital frontal white matter, with the latter correlating with heightened overall impulsivity in attempters [29,34]. This pattern of fronto-limbic disconnection was further supported by findings of reduced FA in the superior longitudinal fasciculus and corona radiata [8,29]. Moreover, disorder-specific fronto-limbic WM alterations included reduced UF FA in BD SAs [51], and decreased white matter integrity in the UF and ventral frontal regions in the attempter group [9].

The corpus callosum (CC), essential for interhemispheric integration, also showed significant microstructural compromise. Studies reported widespread reductions in FA across its subregions (genu, body, splenium) in BD patients with SA compared to healthy controls [8,31,33]. Notably, the severity of these alterations, particularly in the splenium, may have been associated with the intensity of suicidal intent [33]. Extending these findings, reduced FA has been identified in the middle portion of the forceps minor (FMIN)—a callosal fiber tract connecting prefrontal regions—as well as in the anterior and posterior parts of the right cingulum bundle (CB) in the SA group; FMIN abnormalities further correlated with suicidal ideation and emotional distress at the time of scanning [30].

Cerebellar involvement was highlighted by multiple DTI studies. Reduced FA has been observed in the middle cerebellar peduncles in the BD+SA group [8], linking cerebellar circuitry to the pathophysiological model. Additionally, decreased white matter integrity in the right cerebellum has been reported in attempters [9].

Although the predominant finding was reduced WM integrity, some heterogeneity existed. One study reported no significant group differences in global FA measures. However, within the BD+SA group, higher FA in anterior brain regions was correlated with greater impulsivity [32], suggesting a complex, region-specific relationship between WM structure and clinical traits. Collectively, the DTI literature underscored a pattern of compromised structural connectivity, especially within networks critical for emotional regulation and behavioral control, in BD patients with SA.

### 3.2 Functional Imaging

Sixteen functional neuroimaging studies in BD patients with SA showed a multi-level pattern of dysfunction, encompassing regional activity abnormalities, widespread network dysconnectivity, and altered brain-state dynamics, which collectively pointed towards a failure in the neural systems governing emotion regulation, cognitive control, and self-referential processing.

At the regional level, alterations in spontaneous brain activity were evident. Studies using the amplitude of low-frequency fluctuations (ALFF) and its dynamic variant (dALFF) reported increased activity in limbic and paralimbic regions, including the anterior cingulate cortex (ACC), insula, and temporal areas (including the superior and inferior temporal gyri and temporal pole) in BD patients with SA [3,40,46]. Concurrently, reduced activity was observed in prefrontal control regions such as the inferior frontal gyrus and in sensorimotor areas like the paracentral lobule [3]. Task-based fMRI studies provided convergent evidence, showing increased activation during emotional or cognitive tasks in the amygdala, prefrontal cortex, and cerebellum [49]. Furthermore, aberrant microstructural correlates, indicated by abnormal quantitative T1ρ relaxation times, were found in limbic, basal ganglia, and prefrontal regions in association with suicide attempts [49]. Critically, impaired habituation to repeated stimuli in key cortico-limbic areas—most notably in the amygdala—has been observed in SA and has been associated with higher current depressive and suicidal symptomatology [39]. Adding complexity to regional findings, one study reported that in both BD groups (with and without SA), ALFF was increased in the medial, ventral, and dorsolateral prefrontal cortex (PFC) relative to healthy controls; however, within the BD+SA group, ALFF was decreased in the medial PFC, ventral PFC, and dorsolateral PFC compared to BD+NSA [6].

Network-level analyses revealed profound disruptions in the integration and communication between brain regions. A notable finding was the pattern of hyperconnectivity in the default mode network and hypoconnectivity in the salience network and the cognitive executive network associated with suicide risks [37]. This was reflected in both static and dynamic functional connectivity (FC) measures. SA patients exhibit decreased FC between prefrontal regulatory regions and sensorimotor/thalamic areas [35], as well as between the limbic/subcortical network (encompassing the caudate, nucleus accumbens, and subgenual ACC) and the frontoparietal network; the strength of these connections showed negative correlations with clinical suicide-risk scores [48]. Altered intrinsic connectivity of key hubs like the ventromedial prefrontal cortex (vmPFC) and anterior insula has also been reported, with altered connectivity to orbitofrontal, ventrolateral prefrontal, and cerebellar regions [42]. Dynamic FC analyses further demonstrated increased variability, or instability, in the connections between hippocampal/insula regions and temporal cortices in attempters, which correlated with poorer cognitive performance [36,43].

Further characterizing network dysfunction, a study examining combined SA and NSA groups (SA+NSA) found decreased FC between the bilateral thalamus and frontal cortex, with the most pronounced reduction in the SA group. The same study also reported reduced FC between the bilateral inferior frontal gyrus and both the inferior temporal gyrus and fusiform gyrus; however, this FC was stronger in SA than in NSA. Additionally, the SA+NSA group exhibited decreased FC involving the bilateral amygdala, medial frontal cortex, and ACC [44]. Complementing these findings, compared to NSA, BD patients with SA show increased FC in frontal-temporal regions [50], consistent with the relative strengthening of inferior frontal-temporal connectivity noted above. Moreover, the attempter group exhibits decreased amygdala functional connectivity to the left ventral and right rostral prefrontal cortex. Among attempters, there is a significant negative correlation between right rostral prefrontal connectivity and suicidal ideation, as well as between left ventral prefrontal connectivity and attempt lethality [9].

Emerging research on whole-brain temporal dynamics identified a distinct functional architecture in BD with SA. Those patients demonstrated a higher number of transitions between global brain states. However, the direction of change in dwell time remains unclear. This maladaptive state was characterized by hyperconnectivity between the ACC and the subcortical network [38,45]. Conversely, reduced dynamic FC variability between cognitive-emotional hubs like the dorsal anterior insula and the cerebellum has also been noted [41]. This combination of increased dwell time in a pathological state and reduced whole-brain flexibility may underlie the cognitive and emotional rigidity that predisposes individuals to act on suicidal thoughts.

### 3.3 Quality Assessment

Table 2 presents the NIH quality assessment tool ratings. Thirty studies received a global rating of ‘good’, 7 received a ‘fair’ rating, and none received a ‘poor’ rating.

## 4. Discussion

Structural neuroimaging studies have suggested that suicide attempts in BD are associated with atrophy in the prefrontal cortex (particularly the orbitofrontal and dorsolateral regions), and nuanced changes such as increased gray matter volume (GMV) in the inferior frontal gyrus (IFG) that correlate with impulsivity [20,30,53]. Widespread structural alterations in the corpus callosum were consistently observed, with evidence that some corpus callosum reductions may occur independently of suicidality, while suicidal BD patients showed the smallest regional callosal areas, suggesting a potential vulnerability marker [23,26,28]. Findings regarding the cerebellum were less consistent, although reduced GMV in the cerebellum has been reported in attempters [9]. Those structural deficits, moderated by clinical factors such as illness severity and hospitalization history [25], collectively form a neural substrate that compromises emotional regulation and behavioral inhibition, thereby increasing the risk of transitioning from suicidal ideation to action.

DTI findings indicated that SA in BD are closely associated with widespread disruption of white-matter microstructural integrity. This was most evident within prefrontal-limbic pathways (e.g., the uncinate fasciculus, inferior fronto-occipital fasciculus, and cingulum bundle) and the corpus callosum, structures critical for emotion regulation and interhemispheric coordination [8,31,51]. Reduced fractional anisotropy (FA) has also been observed in the forceps minor, superior longitudinal fasciculus, corona radiata, and middle cerebellar peduncles, with forceps minor abnormalities correlating with suicidal ideation and emotional distress [30]. These connectivity abnormalities may fundamentally contribute to emotional dysregulation and impaired impulse control. The correlations between lower FA in these tracts and higher clinical impulsivity or suicidal intent provided a direct neurobiological link to key behavioral phenotypes of suicide risk [33,54]. Of note, no diffusion weighted imaging (DWI) studies were identified that were eligible for screening in the retrieval process, possibly because DWI-related indices are not well-suited to directly capture the white-matter microstructural changes associated with suicidal behavior in the BD with SA group.

Functional neuroimaging synthesis indicated that SA in BD is associated with a multi-level model of brain dysfunction. At the regional activity level, this involved hyperactivity in limbic-paralimbic regions like the ACC, insula, and temporal areas, alongside hypoactivity in prefrontal control regions [3,40,46]. Notably, one study reported that while both BD groups showed increased ALFF in medial, ventral, and dorsolateral PFC relative to controls, the BD+SA group exhibited decreased ALFF in these regions compared to BD+NSA [6], highlighting the complexity of prefrontal activity alterations. At the network level, it manifested as a disrupted balance among major intrinsic networks—specifically, hyperconnectivity within the default mode network and hypoconnectivity within the salience and cognitive executive networks [37]. This was compounded by functional decoupling between prefrontal regulatory regions and limbic/subcortical areas [35,48], as well as by altered amygdala-prefrontal connectivity that correlated with suicidal ideation and attempt lethality [9]. Furthermore, aberrant fronto-temporal connectivity patterns have been observed, including both decreased and increased connectivity depending on the specific subregions [44,50]. At the level of dynamic properties, the system was characterized by destabilized whole-brain state dynamics, including rigid network patterns, heightened volatility in emotional circuits, and an increased dwell time in a maladaptive “suicide-related” state [38,41,45]. Collectively, these abnormalities represent the dynamic neural signature of emotion dysregulation and impaired cognitive control.

### 4.1 A Multi-Level Neurobiological Model of SA in BD

Convergent evidence from structural and functional neuroimaging studies delineated a coherent, multi-level model of neural dysfunction underpinning SA in BD. This model suggested that SA arises not from a focal brain lesion but from a cascade of failures across hierarchical neural systems, encompassing stable structural vulnerabilities, disconnected white-matter networks, and destabilized functional dynamics [18,55,56]. At the foundational level, gray matter reductions in prefrontal regions critical for top-down regulation—particularly the orbitofrontal and dorsolateral prefrontal cortices—constituted a structural endophenotype of impaired behavioral and emotional control [57,58]. In parallel, reductions in the corpus callosum, which may be partly independent of suicidality but are most pronounced in suicidal patients, pointed to a fundamental interhemispheric integration deficit [59].

This structural compromise, through reduced integrity of white matter pathways, propagates to the level of neural connectivity. DTI consistently has revealed microstructural disintegration within critical white-matter pathways, such as the uncinate and inferior fronto-occipital fasciculi, cingulum bundle, and forceps minor, which functionally tether the prefrontal cortex to limbic structures [59]. This anatomical disconnection likely impairs the efficient transmission of regulatory signals, rendering top-down control ineffective against surges of bottom-up emotional distress. The correlation between lower FA in these tracts and higher clinical impulsivity or suicidal ideation offered a direct link between white-matter pathology and a key behavioral phenotype of suicide risk [30,34].

The functional consequences of this compromised structural and connective scaffold were evident in aberrant brain activity and network organization. Resting-state and task-based fMRI studies suggested a tripartite functional disturbance: localized hyperactivity in nodes like the ACC and temporal lobe, alongside region-specific prefrontal hypoactivity (e.g., within the BD+SA group relative to BD+NSA) [43,60]; a disrupted balance between the default mode (hyperconnected), salience, and central executive networks (hypoconnected) [37]; and altered amygdala-prefrontal as well as fronto-temporal connectivity that correlates with clinical severity [9,44]. Collectively, these functional anomalies represent the real-time neural signature of a brain struggling to regulate emotion, maintain cognitive control, and stabilize its own state—a direct substrate for the affective instability and impulsivity that characterize BD with SA.

### 4.2 An Integrated “Vulnerability-to-Dyscontrol” Cascade

Synthesizing these multi-modal findings allowed for the proposal of an integrated “vulnerability-to-dyscontrol” cascade model for suicidal behavior in BD. The journey from suicidal ideation to action may be conceptualized as a failure at successive neural checkpoints. Initially, the trait-like structural and connective vulnerabilities (e.g., PFC atrophy, corpus callosum alterations, fronto-limbic disconnection, and cerebellar structural compromise) establish a baseline of inefficient emotion regulation circuitry. During mood episodes, this vulnerable system is overwhelmed. Hyperactivity in the ACC and default mode network may fuel unremitting negative self-referential thought and psychic pain, and impaired habituation in the amygdala could perpetuate emotional reactivity [60,61].

The critical transition to action likely occurs when this intense ideation coincides with a failure of the “final brake” systems. The dorsal prefrontal control network, already structurally compromised and functionally decoupled from other regions, fails to inhibit prepotent behavioral impulses [62]. Simultaneously, altered function in the insula and related salience network regions may distort the interoceptive perception and valuation of this distress, lowering the threshold for action [63]. Aberrant fronto-temporal connectivity, including both reduced and increased coupling patterns, may further disrupt the integration of emotional and contextual information, facilitating impulsive decisions. In this context, the suicidal act may emerge as a dysregulated, impulse-driven solution. The model underscores the possibility that SA in BD is not merely a symptom of depression but a potentially distinct neurobehavioral syndrome arising from specific interactions between BD pathophysiology and core suicide-related neural circuitry [64].

Nevertheless, the applicability of the proposed cascade model may be constrained by clinical and methodological heterogeneity in the primary literature. First, the majority of studies did not separately analyze BD-I versus BD-II, and it remains unknown whether the observed prefrontal-limbic structural and connectivity alterations differ by subtype. Second, mood state at scanning varied considerably: some studies included patients in a depressive episode, others in euthymia or mania/hypomania, and several mixed states. Neural correlates of suicide attempt may be state-dependent. For example, limbic hyperactivity might be more pronounced during acute mood episodes [39,65]. Third, medication status differed widely, with some patients on complex regimens including antipsychotics or mood stabilizers that can independently affect gray matter volume and white matter integrity [66,67]. Because primary studies rarely controlled for medication effects, the contribution of pharmacological confounds remains unclear. Given these limitations, the current model should be interpreted as a provisional framework that integrates convergent evidence across heterogeneous samples. Future studies should systematically test model predictions in well-characterized subgroups (e.g., BD-I versus BD-II, depressed versus euthymic, unmedicated samples) and examine whether the cascade holds across different clinical phases and treatment backgrounds.

### 4.3 Limitations and Methodological Challenges

The interpretability of this synthesis was constrained by several significant limitations. First, the overwhelming majority of the selected studies were cross-sectional, severely limiting causal inference. It remained indeterminate whether the observed neural abnormalities are precursors, concomitants, or consequences of suicidal behavior. Second, clinical and methodological differences clouded the findings. Variations in BD subtype, mood state at scan, medication status, and the definition and assessment of suicidal behavior (often conflating SA with non-suicidal self-injury) introduced substantial noise (details in Table 1 & **Supplementary Table 1**). Crucially, most studies lacked adequate psychiatric control groups, making it difficult to disentangle neural correlates specific to suicidality from those associated with BD or acute affective episodes more generally. Third, although the reviewed studies powerfully described group-level differences, their translational utility for individual risk prediction remained minimal. Small sample sizes and a lack of independent replication cohorts hindered the development of reliable neuroimaging biomarkers. Furthermore, the field has yet to fully integrate neuroimaging data with other levels of analysis, such as genetics, systemic inflammation, or detailed phenomenological assessments, which are crucial for a complete understanding of the underlying mechanism.

### 4.4 Future Directions and Translational Implications

Future research may adopt prospective, longitudinal designs tracking high-risk BD individuals (e.g., those with significant suicidal ideation) to establish whether multi-modal neural markers can predict the onset or recurrence of suicidal acts. Studies may rigorously stratify participants by BD phase and suicide attempt characteristics (e.g., lethality, impulsivity) to identify more homogeneous neurophenotypes. Embracing a high-dimensional, integrative approach that combines neuroimaging with other biomarker domains using machine learning techniques is a promising path towards individualized risk stratification.

From a translational perspective, the identified neural circuits offer plausible targets for intervention. Neuromodulation techniques like transcranial magnetic stimulation (TMS) or deep brain stimulation (DBS) could be tailored to modulate hyperactivity in the ACC or hypoactivity in the dlPFC, with neuroimaging serving as a guide for target engagement and a biomarker of treatment response [68]. Ultimately, moving from group-level descriptions to a neuroscience-informed, personalized clinical approach is the paramount challenge and opportunity for preventing suicide in BD.

## 5. Conclusions

Neuroimaging studies have converged to indicate that suicide attempts in bipolar disorder are associated with a distinct pattern of multi-level neural dysfunction. This pattern is characterized by structural and microstructural compromise within prefrontal-limbic and interhemispheric circuits, coupled with a functional profile of limbic hyperactivity, impaired large-scale network balance, and destabilized brain dynamics. Together, these alterations underpin a “vulnerability-to-dyscontrol” cascade, wherein inefficient emotion regulation circuits fail to inhibit impulsive behaviors under distress, facilitating the transition from suicidal ideation to action. These neural findings represent potential candidate biomarkers that require prospective validation before they can inform risk prediction or circuit-based interventions for suicide prevention in BD.

## Data Availability

The datasets generated and analyzed during the current study are available from the corresponding authors on reasonable request.
