# Supplementary material for: Neuroimaging Studies of Bipolar Disorder With Suicide Attempts: A Systematic Review
Source: Alpha Psychiatry. 2026 Sep 10;27(5):50801. doi: 10.31083/AP50801 (PMC13732002; doi:10.31083/AP50801)
Supplement: Supplementary file 1 [file 2757-8038-27-5-50801-s1.zip › Supplementary Material/Supplementary Table 1.docx]

**Supplementary Table 1**

| **Authors and year** | **Study design** | **Description of suicide attempt** | **Medication status** | **Covariates used** | **Multiple-comparisons correction (threshold)** |
| --- | --- | --- | --- | --- | --- |
| [Huang et al.(2023)](http://webofscience-clarivate-cn-s.webvpn.zju.edu.cn:8001/wos/author/record/8971308) | sMRI | A patient who stated having committed one or more acts of self-injury with intent to die | medicated: antipsychotics, antidepressants, mood stabilizers | Age, sex, MADRS  score, YMRS score, psychotropic medication use, eTIV | Benjamini–Hochberg false discovery method  (p < 0.05) |
| Villa et al.(2023) | sMRI | A history of past suicide attempts was assessed either via the SCID or via the Columbia Suicide History Form | 84 medicated | Age, gender, scanner type | FWE (p < 0.05) & Bonferroni correction  (p < 0.05) |
| Jabbi et al.(2020) | sMRI | a suicide attempt history was extracted from interviewer notes, but SA lacked a clear definition | unmedicated | Age, sex, TIV | uncorrected (p < 0.001) |
| [Huber et al.(2019)](http://webofscience-clarivate-cn-s.webvpn.zju.edu.cn:8001/wos/author/record/15956511) | sMRI | reported a history of one or more deliberate SA with the intent to die | 22 medicated: antidepressant, mood stabilizer | Medication use, age, sex | FDR correction  (p < 0.1) |
| Gifuni et al.(2017) | sMRI | SA defined as any intentional attempt to die associated with a significant self-inflicted injury | 99 medicated: antipsychotics, lithium, anticonvulsant, anxiolytics, hypnotics | / | Bonferroni correction  (p < 0.05) |
| Duarte et al.(2017) | sMRI | reported one or more self-directed injurious acts with a variable degree of intent to end one's own life | medicated: lithium, anticonvulsants, antidepressant, atypical antipsychotics, benzodiazepine, non benzodiazepine hypnotics | Total brain GMV, age, sex and education | SVC &  FWE-correction  (p < 0.05) |
| [Lijffijt et al.(2014)](http://webofscience-clarivate-cn-s.webvpn.zju.edu.cn:8001/wos/author/record/29214693) | sMRI | self-injurious act with lethal intent | 51 medicated: 8 Lithium | Scanner type, age | uncorrected (p < 0.05) |
| [Nery-Fernandes et al.(2012)](http://webofscience-clarivate-cn-s.webvpn.zju.edu.cn:8001/wos/author/record/26570695) | sMRI | reported one or more self-injurious acts committed with the intent to die | medicated:mostly mood stabilizers | / | uncorrected (p < 0.05) |
| [Baldaçara et al.(2011)](http://webofscience-clarivate-cn-s.webvpn.zju.edu.cn:8001/wos/author/record/419677) | sMRI | reported one or more self-injurious acts committed with the intent to die | / | / | uncorrected (p < 0.05) |
| [Matsuo et al.(2010)](http://webofscience-clarivate-cn-s.webvpn.zju.edu.cn:8001/wos/author/record/47687964) | sMRI | reported one or more self-injurious acts committed with at least some intent to die | 12 medicated: antidepressants, mood stabilizers, atypical antipsychotics | TBV, age | uncorrected (p < 0.05) |
| [Vishwakarma et al.(2025)](http://webofscience-clarivate-cn-s.webvpn.zju.edu.cn:8001/wos/author/record/17089242) | DTI | patients with more than one lifetime SA across discrete mood episodes, with at least one SA in the previous year | mood stabilizer, lithium, valproate | / | GRF (cluster-p < 0.001) & FDR (p < 0.05) |
| [Jiang et al.(2022)](http://webofscience-clarivate-cn-s.webvpn.zju.edu.cn:8001/wos/author/record/9504943) | DTI | defined as a self-destructive act with the purpose to die at least one attempt in one's lifetime | 32 medicated: antipsychotics, antidepressants, mood stabilizers | Age, sex, medication | GRF (voxel-p < 0.005, cluster-p < 0.05) & Bonferroni correction  (p < 0.05) |
| Lima Santos et al.(2021) | DTI | defined as self-injurious acts that met criteria of at least minimal intent (score of 2 or above) and mild medical lethality (score of 3 or above) | medicated: antidepressants, lithium, nonlithium mood stabilizers, antipsychotics, stimulants, benzodiazepines | / | FDR correction  (p < 0.05) |
| [Wei et al.(2020)](http://webofscience-clarivate-cn-s.webvpn.zju.edu.cn:8001/wos/author/record/30936001) | DTI | Prior SA was defined as history of at least one attempt defined as a self-destructive act with some degree of intent to die | 103 medicated: antidepressants, antipsychotics, mood stabilizer | Age, sex | GRF (p < 0.001) |
| Reich et al.(2019) | DTI | A patient was considered to have made an attempt if he or she had at least one deliberate SA, which excluded ambiguous, aborted and interrupted attempts | medicated: antipsychotics, mood-stabilizers | / | uncorrected (p < 0.05) |
| Cyprien et al. (2016) | DTI | defined as any non-fatal,self-directed potentially harmful behavior with the intent to die as a result | medicated: antidepressant, lithium, anticonvulsant, antipsychotics, anxiolytics | Age, NART score, the FA values of the different CC regions, SIS total score, age, number of suicide attempts | FDR correction  (p < 0.025) |
| Mahon et al. (2012) | DTI | reported having ever committed one or more self-injurious acts that were intended to result in death. | 25 medicated: antipsychotics, mood-stabilizers | Age | FWE (p < 0.025) |
| [Huang et al.(2025)](http://webofscience-clarivate-cn-s.webvpn.zju.edu.cn:8001/wos/author/record/8971308) | fMRI | SA was defined as any self- injurious act with intent to die, as reported by the patient. | antipsychotics, antidepressants, mood stabilizers | psychotropics | Benjamini–Hochberg false discovery method  (p < 0.05) |
| [Zhong et al. (2024)](http://webofscience-clarivate-cn-s.webvpn.zju.edu.cn:8001/wos/author/record/18621509) | fMRI | a history of SA within 1 month before enrollment, but SA lacked a clear definition | unmedicated | Age, gender, mean FD | GRF (voxel-p < 0.001, cluster-p < 0.0125) & Bonferroni correction  (p < 0.05) |
| [Wei et al.(2024)](http://webofscience-clarivate-cn-s.webvpn.zju.edu.cn:8001/wos/author/record/43679160) | fMRI | prior suicide attempted history | unmedicated in the last 2 weeks | / | FDR (p < 0.05) |
| [Wang et al.(2024)](http://webofscience-clarivate-cn-s.webvpn.zju.edu.cn:8001/wos/author/record/30553240) | fMRI | SA was defined as an injurious behavior that does not lead to death, in the presence of irrefutable evidence of a plan to die | unmedicated in the last 2 weeks | / | Bonferroni correction  (p < 0.05) |
| [Vai et al.(2023)](http://webofscience-clarivate-cn-s.webvpn.zju.edu.cn:8001/wos/author/record/1876669) | fMRI | have one or more documented SA (broadly defined as any behavior aimed at killing oneself) during the lifetime | / | Sex, age, medication load | FWE & Bonferroni correction (p < 0.05) |
| Tian et al. (2023) | fMRI | at least one self-injurious act with the intent to die during the recent depressive episode but did not lead to death | unmedicated in the last 6 weeks | Age, gender, years of education, mean FD , HAMD score | uncorrected (p < 0.05) |
| Shunkai et al.(2023) | fMRI | a recent SA (time limit of 1 month) during this episode just before being recruited to this study, but SA lacked a clear definition | unmedicated in the last 6 months | Signals of the brain global mean, white matter, cerebro-spinal fluid, age gender, years of education, the mean FD, depression severity | GRF (voxel-p < 0.005, cluster-p < 0.0125) & Bonferroni correction  (p < 0.05) |
| [Sankar et al.(2022)](http://webofscience-clarivate-cn-s.webvpn.zju.edu.cn:8001/wos/author/record/16439348) | fMRI | the self-injurious act was carried out with some intent to die | 50 medicated: anticonvulsants, antipsychotics, antidepressants, benzodiazepines, stimulants, lithium carbonate, adrenergic agonists,non-benzodiazepine hypnotic , levothyroxine | Age | FWE (voxel-p < 0.001, cluster p < 0.05) |
| Zhong et al.(2022) | fMRI | Patients who had a recent SA during this episode just before being recruited to the study were defined as a SA group | unmedicated | Age, gender, years of education, mean FD | GRF (voxel-p < 0.005, cluster-p < 0.0042) & Bonferroni correction  (p < 0.05) |
| [Zhang et al.(2022)](http://webofscience-clarivate-cn-s.webvpn.zju.edu.cn:8001/wos/author/record/34728946) | fMRI | defined as self-injurious acts committed in the person's lifetime that involved at least some intent to die. | SSRIs, SNRIs, antipsychotics, anticonvulsants, lithium | Age, gender, years of education, antipsychotics, ROIs using antidepressants | GRF (voxel-p < 0.001, cluster p < 0.05) |
| [Wang et al.(2022)](http://webofscience-clarivate-cn-s.webvpn.zju.edu.cn:8001/wos/author/record/28594959) | fMRI | SA referred to at least one documented act of self-injury to die in the current event that is recorded in medical history, and HAMD-17 3-item (suicide) score ≥2 | unmedicated in the last 2 weeks | The signals of the whole brain, white matter, cerebrospinal fluid and Friston 24-parameter | Bonferroni correction  (p < 0.05) |
| Tian et al. (2021) | fMRI | defined as having at least one documented self-injurious act committed with intent to die in current episode | unmedicated in the last 2 weeks | Age, gender, years of education, mean FD , HAMD score | GRF (voxel-p < 0.001, cluster-p < 0.05) & Bonferroni correction  (p < 0.05/3) |
| Gong et al. (2020) | fMRI | SI referred to the desire to kill oneself without taking any practical action. SA is defined as a self-inflicted, potentially injurious behavior with a non-fatal outcome, in the presence of implicit or explicit evidence of intent to die | unmedicated | Age, gender, education, FD values | GRF (cluster-p < 0.05) & Bonferroni correction  (p < 0.05) |
| [Cheng et al.(2020)](http://webofscience-clarivate-cn-s.webvpn.zju.edu.cn:8001/wos/author/record/68546986) | fMRI | defined as non-fatal, self-directed potentially injurious behaviors with some intent to die regardless of whether the behaviors cause injury or not, yet excluding non-suicidal self-injurious behaviors. | anxiolytics, antidepressants, anticonvulsants, lithium, antipsychotics | Friston-24 motion parameters, white matter, cerebrospinal fluid, age, gender, and education level, FD parameters | FWE (p < 0.05) |
| [Wang et al.(2020)](https://pubmed.ncbi.nlm.nih.gov/?term=Wang+H&cauthor_id=32734909) | fMRI | SA referred to at least one documented act of self-injury with the intention of dying in the current event, which is recorded in the medical history, and 17-item HAMD 3-item (suicide) scores ≥2 | unmedicated in the last 2 weeks | Head motion (Friston 24-parameter), the signals of the whole brain, white matter and cerebrospinal fluid, age, gender, education ,mean FD | Bonferroni correction  (p < 0.05) |
| [Shaffer et al.(2022)](http://webofscience-clarivate-cn-s.webvpn.zju.edu.cn:8001/wos/author/record/14250518) | fMRI  quantitative T1ρ | participants attempted suicide at least once | antipsychotics, antidepressants, mood stabilizers, lithium, sedative-hypnotics | Age, mood state | FDR  (p < 0.05) |
| Zhao et al. (2021) | sMRI  fMRI | SA was deffned as a self-destructive act with some degree of intent to end one’s life | 76 medicated: antipsychotics, antidepressants, mood stabilizers, combination | Age, gender, intracranial volume | GRF (voxel-p < 0.001, cluster-p < 0.05) & Bonferroni correction  (p < 0.017) |
| [Jiang et al.(2020)](http://webofscience-clarivate-cn-s.webvpn.zju.edu.cn:8001/wos/author/record/994757) | DTI fMRI | SA was deﬁned if participants had at least one documented self-injurious act with the intent to die in the current episode, conﬁrmed by medical records and the HAMD-17 3-item (suicide) score ≥2 | unmedicated in the last 6 weeks | / | uncorrected (p < 0.05; NBS-p < 0.001) |
| Fan et al.(2019) | sMRI  DTI | defined as self-injurious acts committed with at least some intent to die | 47 medicated: lithium carbonate, anticonvulsants, antipsychotics, antidepressants, stimulants, benzodiazepines, levothyroxine, adrenergic agonists, non-benzodiazepine hypnotic | Age | uncorrected (p < 0.05) |
| [Johnston et al.(2017)](http://webofscience-clarivate-cn-s.webvpn.zju.edu.cn:8001/wos/author/record/23544702) | sMRI  DTI fMRI | SA, deﬁned as self-injurious acts committed with at least some intent to die | 44 medicated: lithium carbonate, anticonvulsants, antipsychotics, antidepressants, stimulants, benzodiazepines, methadone, levothyroxine, adrenergic agonistsadrenergic agonists, naltrexonenon-benzodiazepine hypnotic | Age | AlphaSim (p < 0.05) &  Bonferroni correction  (p < 0.0016) |

**BD:** bipolar disorder; **SA:** suicide attempt; **SI:** suicidal ideation; **TIV:** total intracranial volume; **FDR:** false discovery rate; **NOS:** not otherwise specified; **TBV:** total brain volumes; **GLM:** general linear model; **MADRS:** montgomery-asberg depression rating scale; **YMRS:** young mania rating scale; **eTIV:** estimated total intracranial volume; **GMV:** gray matter volume; **FWE:** family-wise error; **SVC:** small volume correction; **VBM:** voxel-based morphometry; **NART:** national adult reading test; **FA:** fractional anisotropy; **CC:** corpus callosum; **SIS:** the suicide intent scale; **GRF:** Gaussian random field; **NBS:** network-based statistic; **WM:** white matter; **IL-6:** interleukin-6; **FD:** framewise displacement; **HAMD:** Hamilton depression rating scale; **ROIs:** regions of interests.
